# Epidermal Growth Factor Receptor Inhibition with Erlotinib Partially Prevents Cisplatin-Induced Nephrotoxicity in Rats

**DOI:** 10.1371/journal.pone.0111728

**Published:** 2014-11-12

**Authors:** Yukihiro Wada, Masayuki Iyoda, Kei Matsumoto, Yuki Shindo-Hirai, Yoshihiro Kuno, Yasutaka Yamamoto, Taihei Suzuki, Tomohiro Saito, Ken Iseri, Takanori Shibata

**Affiliations:** Division of Nephrology, Department of Medicine, Showa University School of Medicine, Tokyo, Japan; INSERM, France

## Abstract

The effects of blocking the epidermal growth factor receptor (EGFR) in acute kidney injury (AKI) are controversial. Here we investigated the renoprotective effect of erlotinib, a selective tyrosine kinase inhibitor that can block EGFR activity, on cisplatin (CP)-induced AKI. Groups of animals were given either erlotinib or vehicle from one day before up to Day 3 following induction of CP- nephrotoxicity (CP-N). In addition, we analyzed the effects of erlotinib on signaling pathways involved in CP-N by using human renal proximal tubular cells (HK-2). Compared to controls, rats treated with erlotinib exhibited significant improvement of renal function and attenuation of tubulointerstitial injury, and reduced the number of apoptotic and proliferating cells. Erlotinib-treated rats had a significant reduction of renal cortical mRNA for profibrogenic genes. The Bax/Bcl-2 mRNA and protein ratios were significantly reduced by erlotinib treatment. *In vitro*, we observed that erlotinib significantly reduced the phosphorylation of MEK1 and Akt, processes that were induced by CP in HK-2. Taken together, these data indicate that erlotinib has renoprotective properties that are likely mediated through decreases in the apoptosis and proliferation of tubular cells, effects that reflect inhibition of downstream signaling pathways of EGFR. These results suggest that erlotinib may be useful for preventing AKI in patients receiving CP chemotherapy.

## Introduction

Cisplatin (CP) is a frontline chemotherapeutic agent used in the treatment of solid tumors [1], [2]. An important side effect of CP administration is acute kidney injury (AKI); approximately one-third of patients show evidence of renal dysfunction following CP treatment [3], [4]. In the kidneys, renal tubular cells are particularly sensitive to CP treatment. Depending on its concentration, CP induces necrosis as well as apoptosis in these cells, leading to AKI [4], [5]. Therefore, finding an effective way to prevent CP-induced AKI is a critical issue.

Erlotinib (TARCEVA, Cugai Pharmaceutical/F. Hoffmann-La Roche, Basel, Switzerland), a selective tyrosine kinase inhibitor that inhibits the epidermal growth factor receptor (EGFR), has been demonstrated to be highly active in patients with non-small cell lung cancer, pancreatic cancer, and several other types of cancer [6]. In general, EGFR is displayed on the cell surface, where the receptor is activated by binding of its ligands, including EGF, heparin-binding EGF-like growth factor (HB-EGF), transforming growth factor-α (TGF-α), amphiregulin, betacellulin, epiregulin, and the neuregulins [7], [8]. Upon activation by its growth factor ligands, EGFR autophosphorylates at several tyrosine residues. This autophosphorylation elicits downstream activation and signaling. This downstream signaling initiates several signal transduction cascades, principally the mitogen-activated protein kinase (MAPK) and the phosphoinositide 3-kinase (PI3K)-Akt pathways, which act to regulate cellular processes such as proliferation, apoptosis, migration, and differentiation [8]–[10].

In the kidney, EGFR is expressed in tubular cells and glomerular podocytes [8], [11], [12]. HB-EGF is expressed in tubular cells, but not in glomeruli [13]. In previous studies, the HB-EGF–EGFR cascade was shown to modulate the proliferation and migration of tubular epithelial cells, promoting the epithelial regeneration response to AKI [14], [15]. However, in contrast to those reports, other studies suggest that inhibition of the HB-EGF–EGFR cascade might be useful in preventing the occurrence and progression of severe renal damage [14], [16]. Therefore, the involvement of EGFR tyrosine kinase activation and therapeutic effects of EGFR pharmacological inhibition in kidney diseases should be studied further.

Although many drugs with different mechanisms of action have been tested to determine whether they could ameliorate or prevent experimental CP-induced nephrotoxicity (CP-N) [4], [17], erlotinib has not been tested. In addition, renoprotective effects of erlotinib on AKI are controversial [8], [18]. Therefore, we investigated the preventive effect of erlotinib in CP-induced AKI, and determined whether erlotinib affects renal tubular cell proliferation and apoptosis. In the present study, we show that erlotinib has preventive effects in experimental CP-N; this preventive role is mediated by reductions in tubular cell proliferation and apoptosis, but not by reductions in inflammation in tubules.

## Materials and Methods

### Experimental Protocol

The experimental protocol for this study was reviewed and approved by the Animal Care Committee of Showa University in Tokyo (Permit number: 03068). Six-week-old male Sprague-Dawley (SD) rats weighing 180 to 210 g were purchased from Sankyo Labo Service Corporation, Inc. (Tokyo, Japan), for use in all of the experiments. The animals were housed in the animal care facility of Showa University under standard conditions (25°C, 50% humidity, 12-hour dark/light cycle) with free access to food and water.

**Figure 1:**
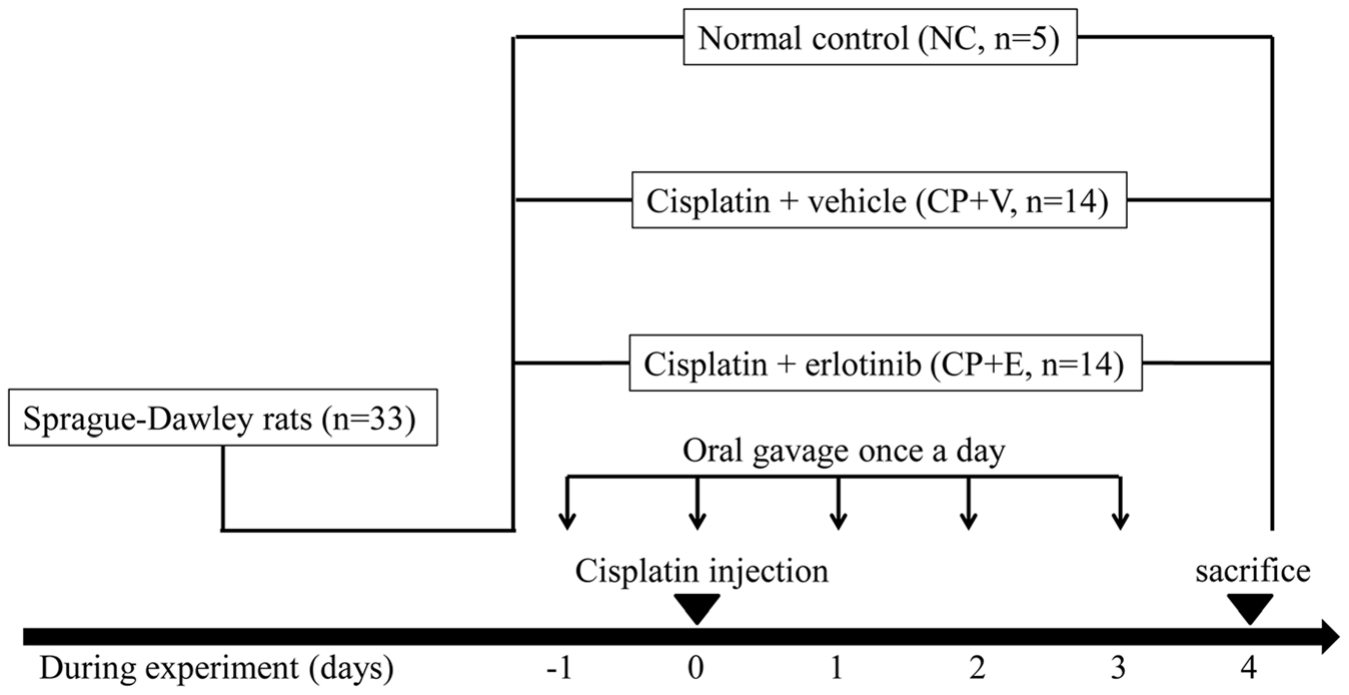
Schematic representation of experimental protocol. Cisplatin (CP) nephrotoxicity was induced in 6-week-old male Sprague-Dawley (SD) rats by intraperitoneal injection of CP on day 0. Groups of animals were administered either erlotinib (CP+E) or vehicle (an equivalent volume of saline) (CP+V) daily by oral gavage from day -1 to day 3. An additional five male SD rats were used as normal controls (NC). At 4 days after CP injection, all rats were sacrificed.

CP (Sigma-Aldrich, St. Louis, MO, USA) was freshly prepared in saline at a concentration of 1 mg ml^−1^ and then injected intraperitoneally in SD rats (n = 28) at a dose of 7 mg/kg on day 0. The dose of CP was selected based on a previous study [19]. To investigate the effect of erlotinib, 28 CP-N rats were divided into two groups. Separate groups (n = 14) each of animals were administered with either erlotinib (20 mg/kg, Cugai Pharmaceutical/F. Hoffmann-La Roche, Basel, Switzerland) (CP+E, n = 14) or vehicle (CP+V, n = 14) daily by oral gavage from day -1 (24 hours prior to the CP injection) to day 3. Vehicle-treated groups received an equivalent volume of saline. Five male SD rats at the age of 6 weeks were used as a normal control group (NC, n = 5). The NC rats were given an equivalent volume of saline daily by oral gavage from day -1 to day 3. At day 4 (96 hours after CP injection), each rat was anesthetized and sacrificed by exsanguination after the cardiac puncture; blood was collected by cardiac puncture and kidneys were collected (Figure 1). Renal tissue was divided; separate portions were snap-frozen in liquid nitrogen or fixed in 2% paraformaldehyde/phosphate-buffered saline (PBS) for later use. All surgery was performed under diethyl ether gas anesthesia, and all efforts were made to minimize suffering.

### Assessment of Biochemical Parameters

Body weight (BW) was recorded periodically, and Kidney weight (KW) was recorded at sacrifice. Serum creatinine (s-Cr), blood urea nitrogen (BUN), and urinary Cr were measured by standard methods using a FUJI DRI-CHEM 3500i (Fuji Photo Film, Tokyo, Japan). For the urinary analysis, rats were housed individually in metabolic cages for 24-h urine collection. Cr clearance (Ccr) was calculated using a standard formula. Urinary N-acetyl-β-D-glucosaminidase (NAG) activity was measured using a commercially available assay kit according to the manufacturer's instructions (Diazyme, General Atomics, San Diego, CA, USA) and expressed as units per liter, and the Urinary NAG index was calculated using the following equation: NAG index (U/g Cr) = urinary NAG activity (U/L)/urinary creatinine concentration (g/L).

### Light Microscopic Study

Tissues fixed in 2% PBS were embedded in paraffin using routine protocols. Paraffin-embedded materials were sectioned at 1-µm thickness for routine staining with periodic acid-Schiff (PAS) and Masson trichrome. Luminal hyaline casts were assessed in 20 fields for each PAS-stained section. The number of casts was counted under x 200 magnification, and the mean number per field was calculated. For evaluating renal tubular injury, 25 fields from each Masson trichrome-stained section were evaluated under x200 magnification. The extent of tubular injury was assessed by counting the percentage of areas with tubular dilatation, tubular cast formation, and tubular epithelial cell necrosis per field of cortex. Scores from 0 to 5 were used: 0, none; 1, <10%; 2, 10–25%; 3, 25–50%; 4, 50–75%: 5, >75% of areas injured, and the results were averaged.

### Immunohistochemistry

The antibodies used in this study were as follows: mouse monoclonal anti-rat ED1 antibody (BMA, Augst, Switzerland) as a macrophage marker, mouse monoclonal anti- proliferating cell nuclear antigen (PCNA) antibody (Progen, Heidelberg, Germany) as a proliferating cell marker, and rabbit polyclonal anti-active caspase-3 antibody (Promega Corporation, Madison, WI, USA) as a marker of apoptosis. EnVisionTM+Dual Link System HRP, based on HRP-labeled polymer, was purchased from Dako (Glostrup, Denmark). Immunohistochemical staining for ED1 (1∶50 antibody dilution), PCNA (1∶10 antibody dilution), and caspase-3 (1∶250 antibody dilution) was performed as follows: the paraffin sections of renal tissues were dewaxed and washed in PBS. H2O2 (0.3%) was added to slides for 30 min for the quenching of endogenous peroxidase. Sections were washed in PBS, treated with protein blocking solution, and incubated overnight at 4°C with the anti-ED1, anti-PCNA, and anti-active caspase-3 antibodies as the primary antibodies. Sections were pretreated twice for 5 min in a conventional household microwave (500 W; Sharp, Osaka, Japan) for ED1 staining or autoclave-heated at 121°C for 10 min for PCNA staining before application of the primary antibodies. Sections were washed thrice in PBS, then incubated with EnVisionTM+Dual Link System-HRP (Dako) for 40 min at room temperature. Next, the sections were developed using diaminobenzidine (DAB) (Dako) as the substrate to produce a brown stain, and sections were counterstained with hematoxylin for ED1 and PCNA staining and with methyl green for caspase-3 staining. Cells undergoing apoptosis were identified by in situ terminal deoxynucleotidyl transferase (Tdt)-mediated dUTP nick end labeling (TUNEL) using the ApopTag plus peroxidase in situ apoptosis detection kit (Chemicon International Inc., Temecula, CA, USA).

The quantification of PCNA, ED-1, TUNEL, and caspase-3-positive cells in the tubulointerstitium was performed by counting the number of positively stained cells in 25 consecutive renal cortical fields under x 400 magnification, and the values were averaged per field.

### Real-time Reverse Transcription Polymerase Chain Reaction

Expression of rat genes encoding IL-6, IL-1β, TNFα, IL-10, Bax, Bcl-2, TGF-α, TGF-β, collage type I, collagen type III, pro-HBEGF, and glyceraldehyde-3-phosphate dehydrogenase (GAPDH) were analyzed using real-time reverse transcription polymerase chain reaction (RT-PCR) in kidney tissues (cortex) as described previously [20]. mRNA expression was normalized using GAPDH as an endogenous control to correct for the differences in the amount of total RNA added to each reaction.

### Western Blot Analysis

Sixty micrograms of protein in kidney tissue homogenate from each sample was separated on a 4–20% gradient gel (Invitrogen) using SDS-PAGE and transferred to PVDF membrane. The blots were blocked with TBST buffer [20 mM Tris-HCl (pH 7.4), 140 mM NaCl and 0.05% Tween 20] containing 5% skimmed milk at room temperature for 1 h, washed three times in TBST buffer and incubated with primary antibody [Bax (Cell Signaling Technology, 1∶1000), Bcl-2 (Cell Signaling Technology, 1∶1000), and GAPDH (Cell Signaling Technology, 1∶3000)] overnight at 4°C. The membranes were then incubated with secondary antibody [HRP-conjugated anti-rabbit IgG antibody (Cell Signaling Technology, 1∶3000)] at room temperature for 1 h. The reaction products were detected using the enhanced chemiluminescence detection system. Changes in Bax and Bcl-2 expression were normalized by correction for the denstiometric intensity of GAPDH for each sample.

### Human Proximal Tubular Cell Culture

Human proximal tubular cells (HK-2 cells) that exhibit biochemical and morphological features of normal proximal tubular cells in culture were obtained from the American Type Culture Collection (ATCC, Manassas, VA, USA). Cells were grown in DMEM containing 10% fetal bovine serum (FBS), a 1% streptomycin-penicillin mixture, 44 mM NaHCO3, and 14 mM HEPES in an atmosphere of 5% CO2 and 95% air at 37°C in a humidified incubator. Experiments were performed with cells up to the fifth passage, as it has been shown that there are no phenotypic changes up to this passage number [21].

### Bio-Plex Suspension Array System

HK-2 cells were initially cultured for 48 hours in medium containing 10% FBS, and then cultured overnight in medium containing 2% FBS. Cells were preincubated for 3 hours with erlotinib (5 µM in dimethylsulfoxide, DMSO) or with DMSO vehicle control, followed by stimulation with medium or CP (8 µg/ml) for 20 min. Cells then were washed with PBS, and total protein was extracted using M-PER Mammalian Protein Extraction Reagent (Pierce Biotechnology, Rockford, IL) containing 1% (vol/vol) protease inhibitor cocktail (Sigma) and 1% (vol/vol) phosphatase inhibitor cocktail (Sigma). Harvested lysates then were centrifuged for 10 min at 4°C to remove cellular debris. The supernatants were collected and stored at -80°C. Protein concentration was measured using the BCA protein assay reagent kit (Pierce Biotechnology).

The Bio-Plex Suspension Array System (Bio-Rad Laboratories, Hercules, CA, USA) was used according to the manufacturer's instructions. This system permitted simultaneous quantification of multiple phosphorylated and total proteins (specifically, phospho-mitogen-activated protein kinase kinases (MEK) 1, phospho-Akt, total-MEK1, and total-Akt) in a 96-well plate format. A dual-laser, flow-based microplate reader system (Bio-Plex 200, Bio-Rad) was used to detect the fluorescent signal intensity. The relative abundance of the each target protein was reported as the ratio of fluorescence among the wells [22].

### Statistical Analysis

Data are presented as mean ± SEM. A nonparametric Mann-Whitney U-test or a one-way ANOVA following a Tukey post hoc test were performed, and values of P<0.05 were considered statistically significant.

## Results

### Effects of Erlotinib on Biochemical Parameters in CP-N Rats

The experimental design is described in Figure 1. The biochemical parameters are presented in Table 1 for SD rats without CP injection (normal control group, NC), for CP-N-induced rats treated with vehicle (CP+V), and for CP-N-induced rats treated with erlotinib (CP+E).

**Table 1:** Effects of erlotinib on biochemical parameters of the study groups.

	NC	CP+V	CP+E
	(n = 5)	(n = 14)	(n = 14)
% BW gain from initial BW (%)	113.3±0.3	98.2±1.3^**^	105.2±3.5^*#^
Kidney weight (g)	2.24±0.06	2.21±0.06	2.19±0.05
KW/BW ratio (%)	0.88±0.02	0.98±0.03^*^	0.92±0.03
Serum Cr (mg/dL)	0.2±0.1	1.6±0.3^**^	0.8±0.2^**#^
Blood urea nitrogen (mg/dL)	20.8±0.9	67.9±9.9^**^	43.2±7.6^*#^
Cr clearance (ml/min)	3.1±0.7	0.7±0.2^**^	1.6±0.4^*#^
Urine volume (ml/day)	17.2±4.0	13.6±1.7	20.6±2.4^#^
Urinary NAG index (U/g Cr)	19.6±4.8	47.1±3.7^**^	37.6±3.8^*#^

Data are mean ± SEM.

Mann–Whitney test: *P<0.05, **P<0.01, vs. NC; #P <0.05, vs. CP+V.

Abbreviations:NC, normal control rats; CP+V, rats with cisplatin-induced nephrotoxicity treated with vehicle; CP+E, rats with cisplatin-induced nephrotoxicity treated with erlotinib; BW, body weight; KW, kidney weight; KW/BW ratio (%), kidney weight (g) x 100/body weight (g); Cr, creatinine; NAG, N-acetyl-β-D-glucosaminidase.

No mortality was observed in any of the groups throughout the study period. At the time of sacrifice, the NC rats gained BW compared to the initial measurement. In contrast, the CP+V rats lost BW during the experiment, which reflects health deterioration. On the other hand, erlotinib significantly attenuated CP-induced BW loss when compared to the CP+V rats (P<0.05, Table 1). Furthermore, CP+E rats exhibited no adverse effects related to erlotinib such as diarrhea or rash. With regard to the KW, there was no significant difference among the three groups. Consequently, the KW-to-BW (KW/BW) ratio (%) was significantly increased in the CP+V rats compared to the NC rats (Table 1). The CP+E rats showed no significant increase in the KW/BW ratio compared to the NC rats.

In terms of renal function, we evaluated the s-Cr, BUN, and Ccr among the three groups. In addition, urine volume (UV) and urinary NAG index, a marker of tubulointerstitial injury, also were assessed. As shown in Table 1, s-Cr (P<0.01) and BUN (P<0.01) levels were significantly higher, and Ccr (P<0.01) was significantly lower in the CP+V rats than in the NC rats, reflecting marked CP-induced AKI. While UV was not significantly decreased, the NAG level (P<0.05) was significantly increased in the CP+V rats compared to the NC rats. Erlotinib treatment significantly improved renal function in CP-N rats. The CP+E rats showed significant reduction of the levels of s-Cr (P<0.05), BUN (P<0.05), urinary NAG index (P<0.05), and significant increase of UV (P<0.05) and Ccr (P<0.05) compared to the CP+V rats.

### Effects of Erlotinib on Renal Histological Findings in CP-N Rats

**Figure 2:**
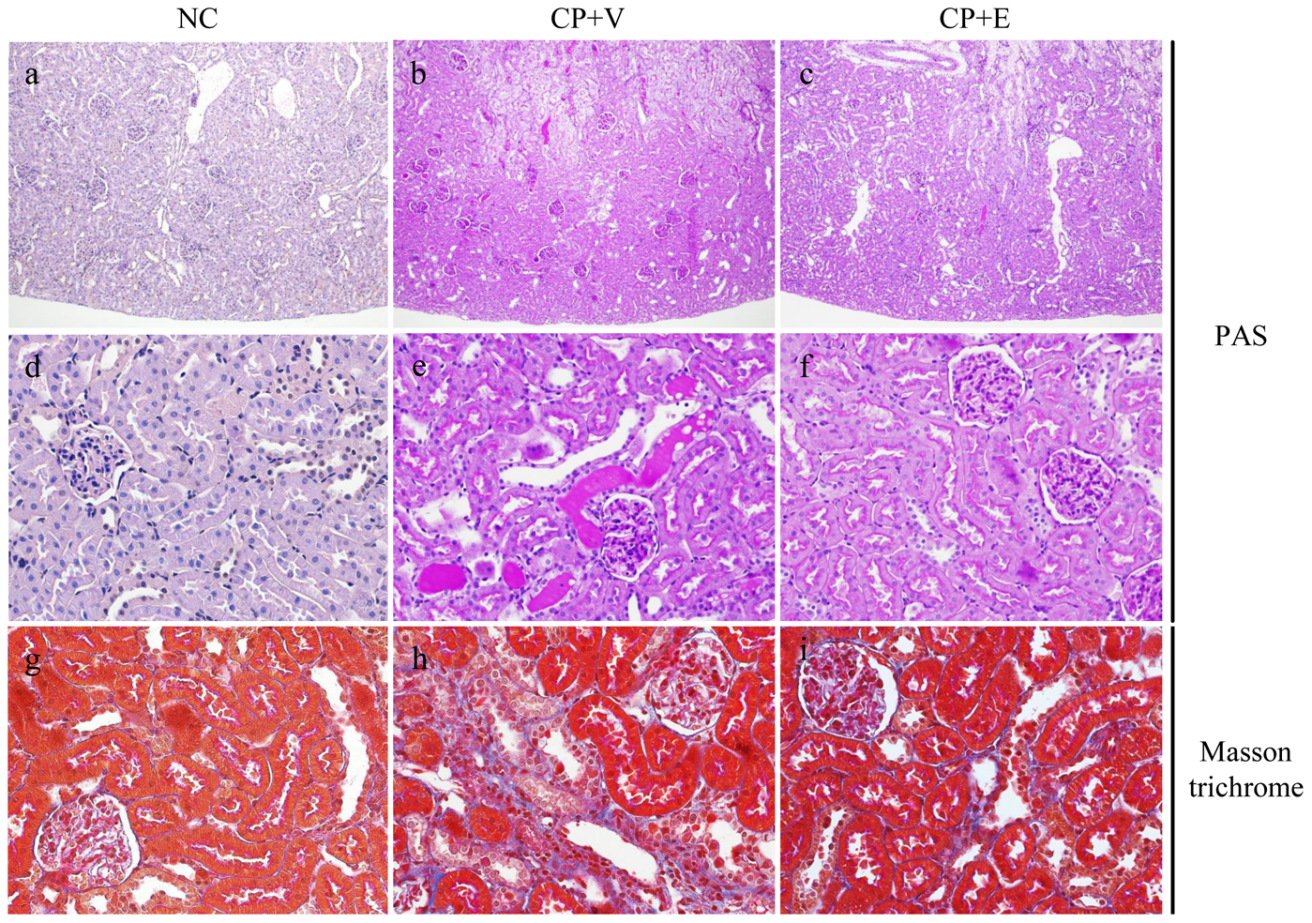
Light microscopic findings in the study groups. Representative images of tissues stained with (a–f) PAS or (g–i) Masson trichrome in a NC rat (a, d, g), a CP+V rat (b, e, h), and a CP+E rat (c, f, i). Original magnifications: (a–c) × 100, (d–i) × 400.

Representative PAS and Masson trichrome stainings of the kidneys from the study groups are shown in Figure 2a–2i. Table 2 shows the results of semiquantitative analysis for casts and tubulointerstitial damage. Compared to the NC rats (Figure 2a, 2d, and 2g), renal histology of the CP+V rats showed severe tubular cell necrosis, loss of brush–border membranes, tubular dilatation, and luminal casts (Figure 2b, 2e, and 2h), and this tubulointerstitial damage was significantly attenuated by erlotinib treatment. Erlotinib treatment significantly reduced the number of luminal hyaline casts (P<0.01) (Figure 2e and 2f, Table 2) and the tubulointerstitial damage score (P<0.01) (Figure 2h and 2i, Table 2) in CP-N rats.

**Table 2:** Morphological evaluation of luminal hyaline casts and tubulointerstitial damage of the study groups.

	NC	CP+V	CP+E
	(n = 5)	(n = 14)	(n = 14)
Casts (number of casts/HPF)	0	2.0±0.7	0.7±0.1^##^
Tubulointerstitial damage score (0–5/HPF)	0	2.4±0.3	1.3±0.1^##^

Data are mean ± SEM.

Mann–Whitney test: ^##^P<0.01, versus CP+V.

Abbreviations:NC, normal control rats; CP+V, rats with cisplatin-induced nephrotoxicity treated with vehicle; CP+E, rats with cisplatin-induced nephrotoxicity treated with erlotinib; HPF, high-power field.

### Effects of Erlotinib on Cell Proliferation, Macrophage Infiltration, and Apoptosis in CP-N Rats

**Figure 3:**
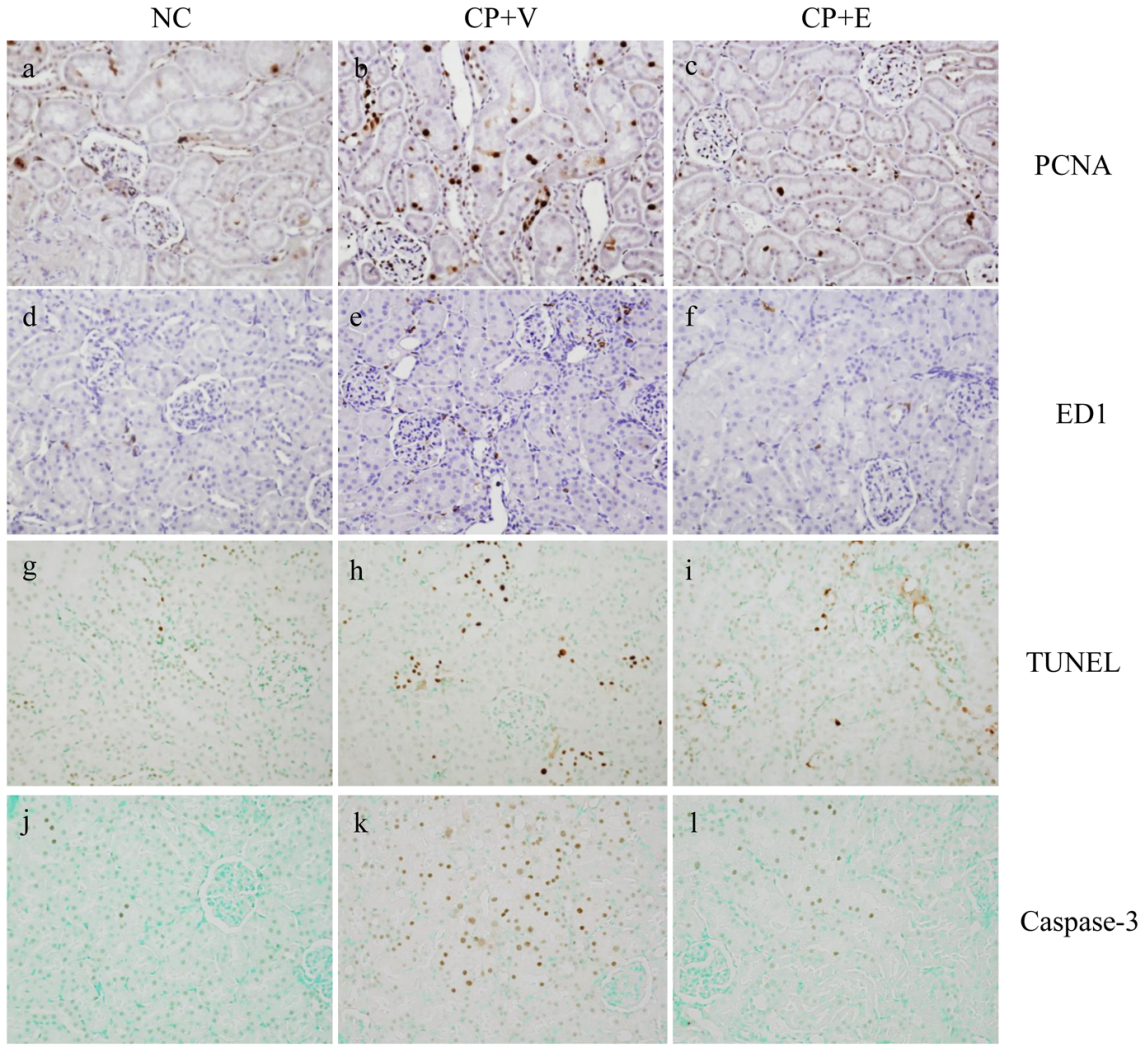
Immunohistochemistry for PCNA, ED1, TUNEL, and caspase-3 in the study groups. Representative pictures stained for (a–c) PCNA, (d–f) ED1, (g–i) TUNEL, and (j–l) caspase-3 in a NC rat (a, d, g, j), a CP+V rat (b, e, h, k), and a CP+E rat (c, f, i, l). Original magnifications: x 400.

Figures 3a–3l show representative images following immunostaining for PCNA (Figure 3a–3c), ED1 (Figure 3d–3f), TUNEL (Figure 3g–3i), and caspase-3 (Figure 3j–3l) in the renal cortex of animals from each group. Table 3 shows the results of quantitative analysis for PCNA, ED1, TUNEL, and caspase-3 staining. Renal tubular cell proliferation was evaluated by staining and quantification of PCNA. The CP+V rats showed a significant increase in the number of PCNA-positive tubular cells compared to the NC rats (P<0.01) (Figure 3a and 3b, Table 3). Erlotinib treatment significantly attenuated the number of PCNA-positive tubular cells in CP-N (P<0.01) (Figure 3b and 3c, Table 3). Quantitative evaluation of tubulointerstitial macrophage infiltration was performed by measurement of ED1-positive cells. As shown in Figures 3d–f and Table 3, there was a significant increase in ED1-positive macrophages in the tubulointerstitium in the CP-N rats compared to the NC rats. No significant difference was detected between the CP+V rats and the CP+E rats (Figure 3e and 3f, Table 3). We evaluated tubular cell apoptosis in the renal cortex by TUNEL and caspase-3 immunostaining (Figure 3g–3l). In the CP+V rats, the number of TUNEL-positive tubular cells was significantly increased compared to that in the NC rats (P<0.01) (Figure 3g and 3h, Table 3). This increased number of apoptotic cells was significantly attenuated by erlotinib treatment (P<0.05) (Figure 3h and 3i, Table 3). Similarly, the increased number of caspase-3-positive cells in CP-N was significantly attenuated by erlotinib treatment (P<0.05) (Figure 3j–3l, Table 3).

**Table 3:** Quantitative evaluation of immunohistochemistry for PCNA, ED1, TUNEL, and Caspase 3 of the study groups.

	NC	CP+V	CP+E
	(n = 5)	(n = 14)	(n = 14)
PCNA, positive cells/HPF	6.7±0.3	23.8±0.6^**^	11.8±0.4^**##^
ED1, positive cells/HPF	3.7±0.4	6.1±0.2^**^	5.7±0.1^*^
TUNEL, positive cells/HPF	4.3±0.2	18.7±0.4^**^	12.6±0.4^**#^
Caspase 3, positive cells/HPF	3.5±0.4	15.5±0.8^**^	12.1±0.7^**#^

Data are mean ± SEM.

Mann–Whitney test: *P<0.05, **P<0.01, versus NC; ^#^P<0.05, ^##^P<0.01, versus CP+V.

Abbreviations:NC, normal control rats; CP+V, rats with cisplatin-induced nephrotoxicity treated with vehicle; CP+E, rats with cisplatin-induced nephrotoxicity treated with erlotinib; HPF, high-power field; PCNA, proliferating cell nuclear antigen; TUNEL, terminal deoxynucleotidyl transferase (Tdt)-mediated dUTP nick end labeling.

### Effects of Erlotinib on Expression of Genes Encoding Fibrogenic Molecules, Proinflammatory Cytokines, Apoptosis-regulatory Molecules, and EGFR ligands in CP-N Rats

**Figure 4:**
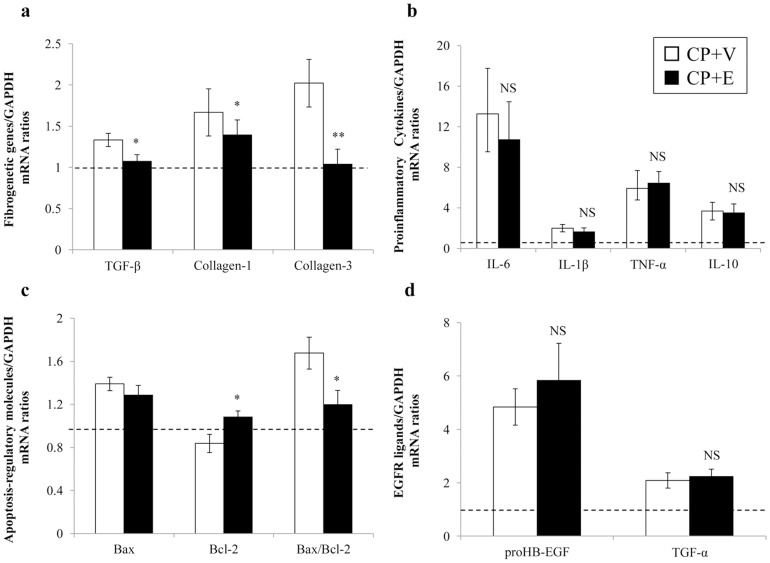
Effects of erlotinib on gene expression levels for fibrogenic molecules, proinflammatory cytokines, apoptosis-regulatory molecules, and EGFR ligands in the study groups. Real-time RT-PCR for genes encoding fibrogenic molecules (a), proinflammatory cytokines (b), apoptosis-regulatory molecules (c), and EGFR ligands including proHB-EGF and TGF-α (d) in each group. The horizontal dotted lines show the expression levels of the NC rats. Data are expressed as mean ± SEM (n = 5, 14, and 14 for the NC rats, the CP+V rats, and the CP+E rats, respectively). The values were normalized to the GAPDH transcript levels and then expressed as relative quantification. Mann–Whitney test: *P<0.05, **P<0.01, NS, not significant vs. CP+V.

Expression of genes encoding profibrogenic molecules TGF-β, collagen type I, and collagen type III were higher in the CP+V rats than in the NC rats, as assessed by RT-PCR (Figure 4a–4d). As shown in Figure 4a, erlotinib treatment significantly decreased TGF-β gene expression in CP-N compared to vehicle treatment (P<0.05). Similar results were obtained for expression of genes encoding collagen type I (P<0.05) and collagen type III (P<0.01) (Figure 4a).

Next, we evaluated expression of the genes encoding proinflammatory cytokines. Expression of the gene encoding IL-1β in the CP+V rats was similar to that in the NC rats (Figure 4b). On the other hand, the expression of the genes encoding IL-6, TNF-α, and IL10 were much higher in the CP+V rats than in the NC rats (Figure 4b). Erlotinib treatment did not affect expression of the genes encoding these proinflammatory cytokines in CP-N rats (Figure 4b).

To assess the effects of erlotinib on apoptosis, we compared renal mRNA expression levels of the genes encoding apoptosis-regulatory molecules in the study groups. As shown in Figure 4c, pro-apoptotic Bax expression level was higher in the CP+V rats than in the NC rats, and this increase was not affected by erlotinib treatment. On the other hand, the expression level of Bcl-2, which encodes an anti-apoptotic protein, was lower in the CP+V rats than in the NC rats, and this decrease was reversed by erlotinib treatment (P<0.05) (Figure 4c). The Bax/Bcl-2 transcript ratio was significantly higher in the CP+V rats than in the NC rats, and this increase was significantly attenuated by erlotinib treatment (P<0.05) (Figure 4c).

We further tested for the effects on the expression of the pro-HB-EGF- and TGF-α-encoding mRNA; pro-HB-EGF and TGF-α correspond to an EGFR-specific ligand. Although pro-HB-EGF mRNA expression levels were higher in the CP+V rats than in the NC rats, no significant difference was seen between the CP+V rats and the CP+E rats (Figure 4d). Similar results were obtained for expression of genes encoding TGF-α (Figure 4d).

### Effects of Erlotinib on Pro-apoptotic and Anti-apoptotic Protein in CP-N Rats

**Figure 5:**
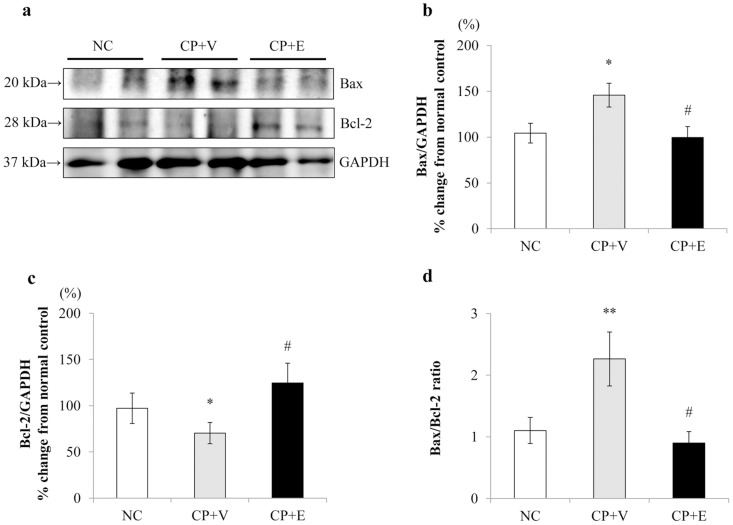
Effects of erlotinib on pro-apoptotic and anti-apoptotic protein in the study groups. Representative western blot analysis for Bax, Bcl-2 and GAPDH (a). Densitometric analysis of western blot for Bax (b), Bcl-2 (c), and Bax/Bcl2 ratio (d) was performed using an image analyzer in each group. Data are expressed as mean ± SEM (n = 5, 14, and 14 for the NC rats, the CP+V rats, and the CP+E rats, respectively). The values were expressed after normalization to GAPDH expression and depicted as the percentage change from the average of normal controls. Mann-Whitney test: *P<0.01, **P<0.01 vs. NC, #P<0.05 vs. CP+V.

To verify the changes in mRNA expression were translated to protein, we evaluated the protein expressions of Bax and Bcl-2 in renal cortex tissue by western blot analysis at 96 hour after CP injection between the three groups. As shown in Figure 5a, western blot analysis demonstrated the faint expression of Bax (20 kDa) and Bcl-2 (28 kDa) in NC rats. The CP+V rats showed a significant increase in Bax protein levels (P<0.05) and a significant reduction in Bcl-2 protein levels (P<0.05) compared with the NC group rats (Figure 5a, 5b, and 5c). Treatment with erlotinib significantly suppressed Bax up-regulation (P<0.05) and reversed the Bcl-2 down-regulation (P<0.05) in CP-N rats. Consequently, the Bax/Bcl-2 ratio was significantly increased in the kidney tissue of the CP+V rats compared to the NC rats (P<0.01), and this increase was significantly attenuated by erlotinib treatment (P<0.05) (Figure 5d).

### Effects of Erlotinib on PI3K-Akt and MAPK Signaling Pathways in Human Proximal Tubular Epithelial Cells

**Figure 6:**
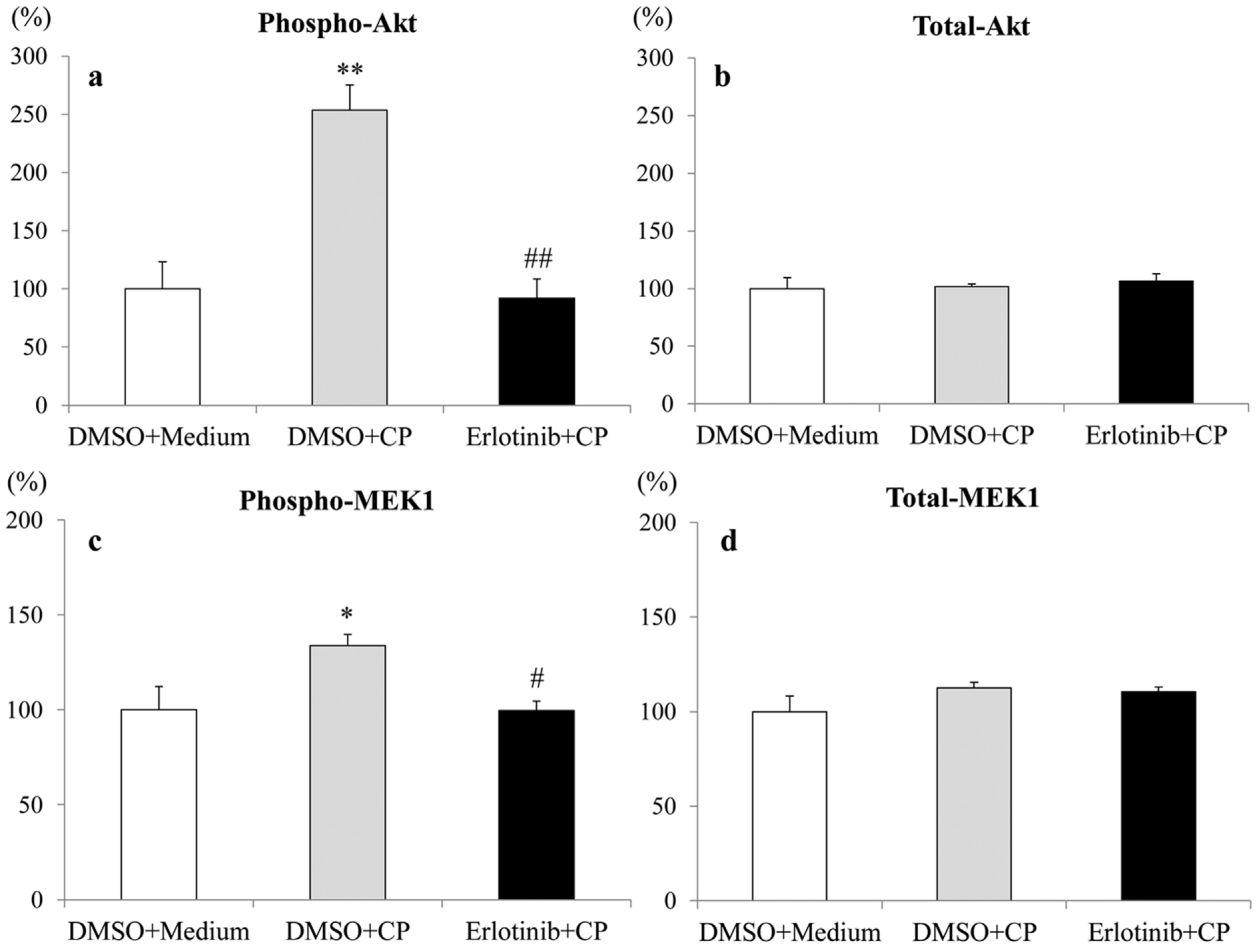
Inhibitory effects of erlotinib on CP-induced PI3K-Akt and MAPK activation in cultured human proximal tubular cells. Phosphorylated Akt (a), total Akt (b), phosphorylated MEK1 (c), and total MEK1 (d) were quantified using the Bio-Plex Suspension Array System. Data are mean ± SEM (n = 4). The values were expressed as the percentage of the mean value of the DMSO+Medium. Mann-Whitney test: *P<0.01 vs. DMSO+Medium, #P<0.05, ## P<0.01 vs. DMSO+CP. Abbreviations: DMSO+Medium, cultures of HK-2 cells without the addition of erlotinib or cisplatin; DMSO+CP, cultures of HK-2 cells stimulated by CP without preincubation with erlotinib; Erlotinib+CP, cultures of HK-2 cells stimulated by CP after preincubation with erlotinib.

To determine the effect of erlotinib on PI3K-Akt and MAPK signaling pathways in HK-2 cells, untreated or CP-stimulated cells were lysed, and Bio-Plex suspension arrays were performed using antibodies against phospho-Akt, total Akt, phospho-MEK1, and total MEK1. As shown in Figure 6a, stimulation with CP increased the levels of phosphorylated Akt; this increase was significantly attenuated by pretreatment with erlotinib in cultured HK-2 cells (P<0.01). On the other hand, no significant difference in total Akt levels was seen among the study groups (Figure 6b). Similarly, stimulation with CP increased the levels of phosphorylated MEK1, and again this increase was significantly attenuated by pretreatment with erlotinib (P<0.01) (Figure 6c); no significant difference was seen in total MEK1 levels among the three groups (Figure 6d).

## Discussion

In this study, we administered erlotinib before the use of CP to evaluate the preventive effect of erlotinib against CP-N. The CP-N rats exhibited renal dysfunction, increased urinary NAG index (representing tubular cell injury), and BW loss. Erlotinib treatment significantly prevented all these manifestations of CP-N. Histopathology, the number of casts, and tubulointerstitial damage also were significantly improved by the treatment with erlotinib. In addition, there was a significant reduction in apoptosis and proliferation of cells in the tubulointerstitium. These data were concomitant with a significant reduction in the renal cortex of the levels of transcripts coding for apoptosis-regulatory and fibrosis-associated molecules. On the other hand, and in accordance with the previous studies [8], we found that erlotinib did not exhibit anti-inflammatory effects. *In vitro*, we demonstrated that pretreatment with erlotinib significantly attenuated the phosphorylation of MEK1 and Akt, events that were induced in proximal tubular epithelial cells by treatment with CP. To the best of our knowledge, this work is the first study to examine the effect of erlotinib in CP-N both *in vivo* and *in vitro*.

In previous studies, an increase in EGFR phosphorylation was detected in the renal proximal tubules in a variety of experimental models of AKI, such as CP-N, ischemia reperfusion, and folic acid administration [23]–[27]. Increased expression of genes encoding EGFR ligands, HB-EGF in particular, also was identified in the kidney after acute tubular injury, including CP-N [23]–[25]. In the present study, we showed that expression of proHB-EGF-encoding mRNA is significantly increased following CP injection. These data suggest that activation of the HB-EGF-EGFR cascade plays an important role in CP-N development. Accordingly, blocking activation of the HB-EGF-EGFR cascade in the proximal tubules (as by erlotinib) is a reasonable therapeutic strategy for treatment of CP-N.

Apoptosis of tubular epithelial cells has been considered as the final common pathway in CP-N, and inhibition of apoptosis is essential for therapeutic strategies for CP-N [4]. In the present study, CP increased the number of TUNEL-positive cells, an effect that was significantly prevented by erlotinib. In the mechanisms of CP-induced tubular cell apoptosis, the role of caspases and Bcl-2 family members have been highlighted in some previous studies [4], [28], [29]. The caspases are a family of cell death proteases involved in the initiation and execution phase of apoptosis. Several studies have demonstrated that CP activates caspase-3, leading to apoptotic cell death in tubular epithelial cells [28]. Members of the Bcl-2 family, including pro-apoptotic proteins such as Bax and anti-apoptotic proteins such as Bcl-2, have been implicated as central regulators of mitochondrial permeability and caspase activation [29]. The ratio of Bax to Bcl-2 has been proposed as an index of the susceptibility of cells to apoptosis, such that an increased ratio indicates predisposition to apoptosis [30]. In the present study, the number of caspase-3-positive cells was significantly attenuated by erlotinib treatment in CP-N rats. Similarly, the elevation of the Bax/Bcl-2 ratio in CP-N was attenuated by erlotinib treatment. Unexpectedly, treatment with erlotinib did not reduce the Bax mRNA expression, erlotinib suppressed Bax protein up-regulation and reversed Bcl-2 mRNA and protein down-regulation in the kidney tissue of CP-N rats. These results indicate that erlotinib exposure might normalize the apoptotic pathways in CP-N rats.

MEK1/2, as well as extracellular signal-regulated kinases 1 and 2 (ERK1/2), are known to be a key kinase in the MAPK signaling pathway [31], [32]. Previously, it has been reported that not only ERK1/2 but also MEK1/2 are deeply involved in CP-induced tubular cell apoptosis [4], [33], [34]. The roles of p38 and Jun N-terminal kinase, which also consist of the MAPK pathway, are less clear in CP-induced tubular cell apoptosis [4], [35]. Jo et al. demonstrated that a MEK inhibitor, U0126, attenuated CP-N by decreasing tubular cell apoptosis in mice [34]. These researchers also demonstrated that U0126 attenuated caspase-3 activation in the kidney tissue of CP-N mice [34]. Similarly, Nowak et al. demonstrated that inhibition of ERK1/2, kinases that are activated following phosphorylation by MEK1/2, attenuated CP-induced caspase-3 activity and apoptosis in cultured renal proximal tubular cells [36]. Moreover, some previous *in vitro* studies indicated that U0126 reduced Bax activation and decreased the Bax/Bcl-2 ratio [35], [37]. Those reports are consistent with our results, in which we confirmed that erlotinib significantly attenuated the CP-induced phosphorylation of MEK1 in tubular epithelial cells. Therefore, we suggest that erlotinib reduces tubular cell apoptosis by inhibition of the EGFR-MAPK signaling pathway. However, our results appear to contrast with those of several other reports. Gao et al. indicated the importance of both the MAPK and PI3K/Akt pathways in CP-induced tubular cell apoptosis [35]. Similarly, Kuwana et al. showed that the PI3K-Akt pathway was activated after CP administration, and noted that blockage of the PI3K/Akt pathway accelerated renal tubular cell apoptosis and led to poor prognoses [38]. Further studies therefore will be necessary to identify the mechanisms behind the activation of the PI3K–Akt pathway in the development of CP-induced tubular cell apoptosis.

The PI3K-Akt pathway is known to regulate the proliferation of tubular epithelial cells [39]–[41]. In previous studies, activation of PI3K-Akt pathway was reported to be required for renal tubular proliferation in primary cultures of renal proximal tubular cells [39]. Additionally, it was demonstrated that the PI3K-Akt pathway, as induced through activation of EGFR, leads to renal tubular proliferation both *in vivo* [41] and *in vitro* [39], [40]. Furthermore, activation of the PI3K-Akt pathway was seen in tubular cells in CP-N mice [38], [42] and in CP-stimulated HK-2 proximal tubular cells [35]. In the present study, we confirmed that erlotinib significantly attenuated CP-induced Akt phosphorylation in cultured proximal tubular epithelial cells. *In vivo*, the number of PCNA-positive tubular cells was significantly reduced by erlotinib treatment. Generally, it is accepted that sustained tubular cell proliferation is closely associated with subsequent development of renal fibrosis [8], [18]. In the present study, we found the erlotinib-treated rats had a significant reduction in the renal cortical expression of genes encoding profibrogenic proteins. Furthermore, recent studies indicated that sustained tubular cell proliferation via activation of EGFR-PI3K-Akt signaling pathway eventually leads to tubulointerstitial fibrosis with elevation of the extracellular matrix protein (ie, collagen type I) in the late phase following ischemic injury [18], although its experimental model was not CP-N. Therefore, we speculate that inhibition of PI3K-Akt pathway by erlotinib might attenuate tubular cell proliferation with subsequent reduction of renal profibrogenic gene expression, thereby leading to the attenuation of CP-N. Further research is necessary to gain more precise understanding of the latter effects (several weeks) of erlotinib on CP-N.

However, there is an opposing notion that the Akt-dependent tubular cell proliferation plays a crucial role in recovery from AKI [15], [41], [42]. He et al. indicated that proliferation of dedifferentiated intrinsic renal tubular cells, mediated by the phospho-EGFR-PI3K-Akt signaling pathway, may be the key step in restoring renal structure and function after acute tubular injury [41]. This discrepancy with our results may reflect differences in experimental protocol (e.g., treatment starting period). We speculate that the role of EGFR-PI3K-Akt signaling pathway for tubular cell proliferation differs between the early and recovery phases of AKI. Recently, there is a growing interest in research that evaluates the combination therapy with erlotinib and CP for patients with advanced cancers, including those of lung, head, neck, and pancreas. Lee et al. demonstrated that the erlotinib-CP combination is an effective treatment against erlotinib-resistant cancer cells [43]. Similarly, the potential for combining erlotinib with CP has been investigated in several clinical trials [44], [45]. In those studies, erlotinib treatment was initiated before CP therapy, consistent with the experimental protocol described here. Notably, erlotinib did not increase the toxicity of CP in patients with advanced cancers. Of note, no case suffering from severe nephrotoxicity was reported in the erlotinib-CP combination group. The present study supports those clinical studies and suggests a mechanism whereby erlotinib provides protection from CP nephrotoxicity.

With respect to limitations of this study, we must consider several issues. First, food intake was not controlled among the three groups. Since the animals were not pair fed, it was hard to determine whether BW loss was depending on low intake or influence of CP induced AKI itself. Second, the present study did not address tubular dysfunction including salt and magnesium wasting, which is one of the most common physiological abnormalities associated with CP-N. Third, the therapeutic effect of erlotinib on recovery phase from CP-induced AKI was not investigated. Clinically, therapeutic effect on recovery phase as well as preventive effect on early phase is thought to be relevant to patients receiving CP chemotherapy. Lastly, the influence of erlotinib on antitumorigenic effects of CP was not proved. Further studies to evaluate whether the reduction of CP-elicited cell death by erlotinib was specific for the kidney by using different tumor cell lines like previous studies [30], [46] are needed.

In conclusion, our *in vivo* and *in vitro* studies show that erlotinib has a renoprotective effect in CP-N, an effect that might be attributable to the attenuation of the apoptosis and proliferation of proximal tubular cells. Protection by erlotinib appears to be mediated through the inhibition of downstream signaling of EGFR, including MAPK and PI3K-Akt. These results suggest that erlotinib may be useful for preventing AKI in patients receiving CP chemotherapy.

## Supporting Information

Checklist S1 **ARRIVE checklist.**
(DOC)
