# Supplementary material for: Epidermal Growth Factor Receptor Inhibition with Erlotinib Partially Prevents Cisplatin-Induced Nephrotoxicity in Rats
Source: PLoS One. 2014 Nov 12;9(11):e111728. doi: 10.1371/journal.pone.0111728 (PMC4229108; doi:10.1371/journal.pone.0111728)
Supplement: Checklist S1 — ARRIVE checklist. (DOC) [file pone.0111728.s001.doc]

**The ARRIVE Checklist**

**TITLE**

**1 Provide as accurate and concise a description of the content of the article as possible.**

Epidermal growth factor receptor inhibition with erlotinib prevents cisplatin-induced nephrotoxicity in rats

**ABSTRACT**

**2 Provide an accurate summary of the background, research objectives (including details of the species or strain of animal used), key methods, principal findings, and conclusions of the study.**

The effects of blocking the epidermal growth factor receptor (EGFR) in acute kidney injury (AKI) are controversial. Here we investigated the renoprotective effect of erlotinib, a selective tyrosine kinase inhibitor that can block EGFR activity, on cisplatin (CP)-induced AKI. Groups of animals were given either erlotinib or vehicle from one day before up to Day 3 following induction of CP- nephrotoxicity (CP-N). In addition, we analyzed the effects of erlotinib on signaling pathways involved in CP-N by using human renal proximal tubular cells (HK-2). Compared to controls, rats treated with erlotinib exhibited significant improvement of renal function and attenuation of tubulointerstitial injury, and reduced the number of apoptotic and proliferating cells. Erlotinib-treated rats had a significant reduction of renal cortical mRNA for profibrogenic genes. The Bax/Bcl-2 mRNA and protein ratios were significantly reduced by erlotinib treatment. In vitro, we observed that erlotinib significantly reduced the phosphorylation of MEK1 and Akt, processes that were induced by CP in HK-2. Taken together, these data indicate that erlotinib has renoprotective properties that are likely mediated through decreases in the apoptosis and proliferation of tubular cells, effects that reflect inhibition of downstream signaling pathways of EGFR. These results suggest that erlotinib may be useful for preventing AKI in patients receiving CP chemotherapy.

We believe that we are able to provide an accurate summary of the background, research objectives, key methods, principal findings, and conclusions of the study.

**INTRODUCTION**

**Background**

**3 a. Include sufficient scientific background (including relevant references to previous work) to understand the motivation and context for the study, and explain the experimental approach and rationale.**

**b. Explain how and why the animal species and model being used can address the scientific objectives and, where appropriate, the study’s relevance to human biology.**

a) Please refer to the introduction of our manuscript **(Page 4 Lines 11-Page 7, Lines 2).**

b) Although many drugs with different mechanisms of action have been tested to determine whether they could ameliorate or prevent experimental CP-induced nephrotoxicity (CP-N) model (especially rat or mouse model), erlotinib has not been tested. In addition, renoprotective effects of erlotinib on AKI are controversial. Therefore, we investigated the preventive effect of erlotinib in CP-induced AKI by using rat model, and determined whether erlotinib affects renal tubular cell proliferation and apoptosis.

**Objectives**

**4 Clearly describe the primary and any secondary objectives of the study, or specific hypotheses being tested.**

Although many drugs with different mechanisms of action have been tested to determine whether they could ameliorate or prevent experimental CP-induced nephrotoxicity (CP-N), erlotinib has not been tested. In addition, renoprotective effects of erlotinib on AKI are controversial. Therefore, we investigated the preventive effect of erlotinib in CP-induced AKI, and determined whether erlotinib affects renal tubular cell proliferation and apoptosis. In the present study, we show that erlotinib has preventive effects in experimental CP-N; this preventive role is mediated by reductions in tubular cell proliferation and apoptosis, but not by reductions in inflammation in tubules.

**METHODS**

**Ethical statement**

**5 Indicate the nature of the ethical review permissions, relevant licenses (e.g. Animal [Scientific Procedures] Act 1986), and national or institutional guidelines for the care and use of animals, that cover the research.**

The experimental protocol for this study was reviewed and approved by the Animal Care Committee of Showa University in Tokyo (Permit number: 03068).

**Study design**

**6 For each experiment, give brief details of the study design, including:**

**a. The number of experimental and control groups.**

**b. Any steps taken to minimise the effects of subjective bias when allocating animals to treatment (e.g., randomisation procedure) and when assessing results (e.g., if done, describe who was blinded and when).**

**c. The experimental unit (e.g. a single animal, group, or cage of animals).**

**A time-line diagram or flow chart can be useful to illustrate how complex study designs were carried out.**

a) –c) Six-week-old male Sprague-Dawley (SD) rats were purchased for use in all of the experiments. Cisplatin (CP) was freshly prepared in saline at a concentration of 1 mg ml^-1^ and then injected intraperitoneally in SD rats (n = 28) at a dose of 7 mg/kg on day 0.

To investigate the effect of erlotinib, 28 CP-N rats were divided into two groups at random. Separate groups (n = 14) each of animals were administered with either erlotinib (20 mg/kg) (CP+E, n = 14) or vehicle (CP+V, n = 14) daily by oral gavage from day -1 (24 hours prior to the CP injection) to day 3. Vehicle-treated groups received an equivalent volume of saline. Five male SD rats at the age of 6 weeks were used as a normal control group (NC, n = 5). The NC rats were given an equivalent volume of saline daily by oral gavage from day -1 to day 3. At day 4 (96 hours after CP injection), each rat was anesthetized and sacrificed.

**Experimental procedures**

**7 For each experiment and each experimental group, including controls, provide precise details of all procedures carried out. For example:**

**a. How (e.g., drug formulation and dose, site and route of administration, anaesthesia and analgesia used [including monitoring], surgical procedure, method of euthanasia). Provide details of any specialist equipment used, including supplier(s).**

**b. When (e.g., time of day).**

**c. Where (e.g., home cage, laboratory, water maze).**

**d. Why (e.g., rationale for choice of specific anaesthetic, route of administration, drug dose used).**

a)-d) At day 4 (96 hours after CP injection), each rat was anesthetized and sacrificed by exsanguination after the cardiac puncture; blood was collected by cardiac puncture and kidneys were collected (Figure 1). Renal tissue was divided; separate portions were snap-frozen in liquid nitrogen or fixed in 2% paraformaldehyde/phosphate-buffered saline (PBS) for later use. All surgery was performed under diethyl ether gas anesthesia, and all efforts were made to minimize suffering.

**Experimental animals**

**8 a. Provide details of the animals used, including species, strain, sex, developmental stage (e.g., mean or median age plus age range), and weight (e.g., mean or median weight plus weight range).**

**b. Provide further relevant information such as the source of animals, international strain nomenclature, genetic modification status (e.g. knock-out or transgenic), genotype, health/immune status, drug- or test naıve, previous procedures, etc.**

a), b) Six-week-old male Sprague-Dawley (SD) rats weighing 180 to 210 g were purchased from Sankyo Labo Service Corporation, Inc. (Tokyo, Japan), for use in all of the experiments.

**Housing and husbandry**

**9 Provide details of:**

**a. Housing (e.g., type of facility, e.g., specific pathogen free (SPF); type of cage or housing; bedding material; number of cage companions; tank shape and material etc. for fish).**

**b. Husbandry conditions (e.g., breeding programme, light/dark cycle, temperature, quality of water etc. for fish, type of food, access to food and water, environmental enrichment).**

**c. Welfare-related assessments and interventions that were carried out before, during, or after the experiment.**

a), b) The animals were housed in the animal care facility of Showa University under standard conditions (25°C, 50% humidity, 12-hour dark/light cycle) with free access to food and water.

c) Not applicable

**Sample size**

**10 a. Specify the total number of animals used in each experiment and the number of animals in each experimental group.**

**b. Explain how the number of animals was decided. Provide details of any sample size calculation used.**

**c. Indicate the number of independent replications of each experiment, if relevant.**

In order to evaluate the effect of erlotinib on CP-N rats, we performed cohort study twice. First, 15 SD rats (6-week-old, male) were randomly divided into CP+V group (n=7) and CP+E group (n=8). CP-N was induced in 15 SD rats by i.p injection of CP (7 mg/kg) on day 0. Groups of animals were given either erlotinib (20 mg/kg) or vehicle (equal volume of saline) daily by oral gavage from one day before up to Day 3. In that investigation, we obtained preliminary data that CP+E rats showed significantly reduction of sCr level compared to CP+V rats (0.7 ± 0.2 vs 1.2 ± 0.4 mg/dl, P=0.0163). Thus, we performed the second investigation with same protocol except for the dividing (n=7 for CP+V rats, n=6 for CP+E rats, respectively). In addition, 5 SD rats were used as normal control. In the second investigation, we obtained the similar results (sCr level: 0.9 ± 0.5 vs 1.9 ± 0.3 mg/dl, P=0.0432). Finally, we reached the conclusion, in which erlotinib attenuates cisplatin-induced nephrotoxicity.

**Allocating animals to experimental groups**

**11 a. Give full details of how animals were allocated to experimental groups, including randomisation or matching if done.**

**b. Describe the order in which the animals in the different experimental groups were treated and assessed.**

a) Experimental rats were divided into experimental groups (at random).

b) All rats from each study groups were investigated simultaneously.

**Experimental outcomes**

**12 Clearly define the primary and secondary experimental outcomes assessed (e.g., cell death, molecular markers, behavioural changes).**

At day 4 (96 hours after CP injection), all rat among the study groups were anesthetized and sacrificed by exsanguination after the cardiac puncture; blood was collected by cardiac puncture and kidneys were collected for further evaluation.

**Statistical methods**

**13 a. Provide details of the statistical methods used for each analysis.**

**b. Specify the unit of analysis for each dataset (e.g. single animal, group of animals, single neuron).**

**c. Describe any methods used to assess whether the data met the assumptions of the statistical approach.**

a)-c) Data are presented as mean ± SEM. A nonparametric Mann-Whitney U-test or a one-way ANOVA following a Tukey post hoc test were performed, and values of P<0.05 were considered statistically significant.

**RESULTS**

**Baseline data
14 For each experimental group, report relevant characteristics and health status of animals (e.g., weight, microbiological status, and drug- or test-naıve) before treatment or testing (this information can often be tabulated).**

All animals analyzed were in good health at baseline (Day 0)

**Numbers analysed**

**15 a. Report the number of animals in each group included in each analysis. Report absolute numbers (e.g. 10/20, not 50%).**

**b. If any animals or data were not included in the analysis, explain why.**

The number (n) for Table 2-3 and Figure 4 is same as Table 1. The number of rats is as follows: n=5, 14, and 14 for the NC rats, the CP+V rats, and the CP+E rats, respectively.

**Outcomes and estimation**

**16 Report the results for each analysis carried out, with a measure of precision (e.g., standard error or confidence interval).**

Please refer to our manuscript **(Page 16, Lines 2-Page 23, Lines 8)** and Table 1-3, Figure 2-6.

**Adverse events**

**17 a. Give details of all important adverse events in each experimental group.**

**b. Describe any modifications to the experimental protocols made to reduce adverse events.**

We observed not only mortality but also adverse effects of all rats during the study period. The CP+E rats exhibited no adverse effects related to erlotinib such as diarrhea or rash throughout the study period.

**DISCUSSION**

**Interpretation/scientific implications**

**18 a. Interpret the results, taking into account the study objectives and hypotheses, current theory, and other relevant studies in the literature.**

**b. Comment on the study limitations including any potential sources of bias, any limitations of the animal model, and the imprecision associated with the results.**

**c. Describe any implications of your experimental methods or findings for the replacement, refinement, or reduction (the 3Rs) of the use of animals in research.**

Although there were some limitations of this study, the conclusions were thought to be drawn appropriately based on the data presented. Furthermore, we believed that we took into account the study objectives and hypotheses, current theory, and other relevant studies in the literature.

**Generalisability/translation**

**19 Comment on whether, and how, the findings of this study are likely to translate to other species or systems, including any relevance to human biology.**

Our in vivo and in vitro studies show that erlotinib has a renoprotective effect in CP-N, an effect that might be attributable to the attenuation of the apoptosis and proliferation of proximal tubular cells. Protection by erlotinib appears to be mediated through the inhibition of downstream signaling of EGFR, including MAPK and PI3K-Akt. These results suggest that erlotinib may be useful for preventing AKI in patients receiving CP chemotherapy.

**Funding**

**20 List all funding sources (including grant number) and the role of the funder(s) in the study.**

Y.W. and Y.K. were supported by a Showa University Research Grant for Young Researchers. M.I. was supported by the Showa University Medical Foundation. The funders had no role in study design, data collection and analysis, decision to publish, or preparation of the manuscript. No additional external funding received for this study.
